# Knosp and revised Knosp classifications predict non-functioning pituitary adenoma outcomes: a single tertiary center experience

**DOI:** 10.25122/jml-2024-0015

**Published:** 2024-11

**Authors:** Siham Rouf, Soumiya Berrabeh, Lamiae Zarraa, Hanane Latrech

**Affiliations:** 1 Department of Endocrinology, Diabetology and Nutrition, Mohammed VI University Hospital, Medical School, Mohamed the First University, Oujda, Morocco; 2 Laboratory of Epidemiology, Clinical Research, and Public Health, Faculty of Medicine and Pharmacy of Oujda, Mohammed the First University, Oujda, Morocco

**Keywords:** non-functioning pituitary adenomas, Knosp classification, revised Knosp classification, surgical remission

## Abstract

Non-functioning pituitary adenomas (NFPAs) are hormonally inactive benign tumors, usually diagnosed as macro-adenoma. The aim of our research was to analyze the clinical and hormonal characteristics of NFPAs using Knosp and revised Knosp classifications. Furthermore, we aimed to assess the possibility of predicting surgical remission after surgery. This was a prospective descriptive-analytical study. We selected 30 patients with non-functioning pituitary adenomas by excluding all the clinical and biochemical evidence of hormone excess. Cavernous sinus invasion was evaluated by Knosp and revised Knosp classifications. The mean age was 50.8 ± 11.6 years, and 63.3% of the patients were women with a sex ratio F/M of 1.7. Patients with a Knosp grade greater than two experienced more symptoms, such as headaches (*P* = 0.014) and declining visual acuity (*P* = 0.095). Additionally, these patients were found to have a higher prevalence of growth hormone deficiency compared to those with a Knosp grade of two or lower (*P* = 0.037). The revised Knosp classification showed no significant difference between patients with invasive adenomas (grade ≥ 3B) and patients with non-invasive adenomas (grade ≤ 3A) regarding clinical and hormonal status. However, we noticed that patients with non-invasive adenomas (grade ≤ 3A) had significant surgical remission (*P* = 0.008). A preoperative description of cavernous sinus invasion in NFPAs provided by the Knosp and revised Knosp classifications is mandatory. Our report shows that the revised Knosp classification is superior in predicting surgical remission than the Knosp classification, with no significant difference in evaluating the clinical and hormonal status between the two classifications.

## INTRODUCTION

Non-functioning pituitary adenomas (NFPA) are hormonally inactive, non-malignant tumors that arise from the anterior pituitary gland and are not associated with endocrine disorders [1]. They comprise around 80-90% of all well-differentiated pituitary neuroendocrine tumors (PitNETs) [2,3]. Recent epidemiological studies indicate an annual incidence of NFPAs ranging from 0.65 to 2.34 cases per 100,000 individuals, with an estimated prevalence of 7 to 41.3 cases per 100,000 [4,5].

Surgery is considered the gold standard therapy for adenomas, especially NFPAs. It has witnessed significant advancements over time, notably with transsphenoidal surgery considered the gold standard for treating compressive macroadenomas. Attaining surgical remission is an inconstant outcome, and there are no clear predictive clinical or histological factors for non-functioning pituitary adenoma remission after surgery [5-7]. Therefore, preoperative radiological assessment using MRI is mandatory for diagnosing and evaluating the tumor size and invasion degree, which is required to improve postoperative remission prognosis.

The earliest radiographic classification was proposed by Hardy *et al*. in 1976 and modified by Wilson in 1979 [8,9]. Likewise, Knosp *et al*. described the cavernous sinus invasion of pituitary adenomas (PAs) considering the line between the internal carotid artery and intracavernous region on coronal MRI [10]. This classification was revised to differentiate between the inferior and superior invasion of the cavernous sinus in grades 3A and 3B [11]. It is used to predict surgical outcomes of PAs and even endocrine remission in functioning PAs [11,12]. In this study, we aimed to analyze the clinical and hormonal profiles of NFPAs based on these classifications and assess the correlation between these radiological classifications and the prediction of surgical remission after surgery.

## MATERIAL AND METHODS

### Study design and population

This study was conducted at the Department of Endocrinology, Diabetology, and Nutrition. Our approach to managing patients with pituitary adenomas is multidisciplinary and involves endocrinologists, radiologists, neurosurgeons, ophthalmologists, and anatomopathologists. We performed a prospective descriptive-analytical study from January 2016 to December 2021, selecting 30 out of 91 patients admitted for pituitary adenomas, specifically focusing on those diagnosed with non-functioning pituitary adenomas. The selection criteria included:

MRI showing radiological features related to a pituitary adenoma.
Absence of clinical or biochemical hormonal excess, including screening for Cushing’s syndrome, acromegaly, and hyperthyroidism.
Prolactin measurement was conducted with a 1:100 serum dilution to avoid the hook effect, which can mask high levels indicative of prolactinoma.
An immunochemistry analysis was performed to identify hormone secretion and assess the proliferation index (Ki-67) of tissue specimens.
Patients with a complete medical file.

In accordance with international diagnostic criteria, the exclusion of clinical and biochemical evidence of hormonal excess involved screening for Cushing’s syndrome through the overnight dexamethasone suppression test, a 24-hour urinary-free cortisol assessment, and midnight salivary cortisol measurements [13,14]. Additionally, the evaluation included average insulin-like growth factor 1 (IGF1) levels within the reference range, free triiodothyronine (T3) and free thyroxine (T4) levels combined with normal thyroid-stimulating hormone (TSH) levels, and normal follicle-stimulating hormone (FSH)/luteinizing hormone (LH) and estradiol (in women) or testosterone (in men) levels [15].

All patients were evaluated clinically and biochemically for pituitary hormone deficiencies at diagnosis. Growth hormone (GH) testing was performed if GH deficiency was suspected; a response lower than 3-5 µg/l during GH testing confirmed somatotroph deficiency. Screening for corticotroph deficiency required dynamic tests, such as the adrenocorticotropic hormone (ACTH) stimulation test or insulin tolerance test (ITT), with a peak cortisol level lower than 18.1µg/dl (500 nmol/) at 60 minutes suggesting adrenal insufficiency. Thyrotroph deficiency screening involved measuring free thyroxine (FT4) associated with the 3rd generation TSH assay. Gonadotroph deficiency was assessed based on clinical evaluation, and if necessary, it was complemented by gonadotropin and estradiol or testosterone measurements [16].

Transsphenoidal surgery is a treatment option for NFPAs. Surgical indications depend on the presence of neuro-ophthalmologic complications. Visual assessments comprised sensory evaluation with visual acuity and a visual field examination, optical coherence tomography (OCT), and oculomotor evaluation.

All our patients underwent a pituitary MRI respecting a protocol that include T1 weighted sequences with and without gadolinium injection with thin slices (≤ 3mm), sagittal and coronal, and T2-weighted coronal slices with a volume acquisition. An expert radiologist reviewed all the patients’ scans.

The Knosp-Steiner classification was used to evaluate cavernous sinus invasion:

Knosp 0: PA contained within the medial margins of the supra and intracavernous artery (ICA).
Knosp 1: PA extends beyond medial margins.
Knosp 2: PA extends to the space between the lateral tangent and the intercarotid line.
Knosp 3: PA extends beyond lateral margins of the supra and intracavernous artery
Knosp 4: Total encasement of the intracavernous artery.

**Figure 1:**
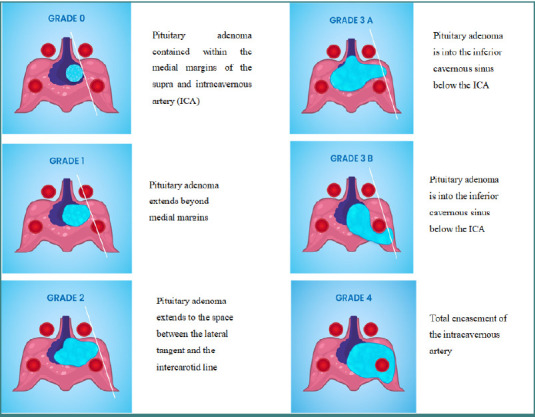
Revised Knosp classification separates grades 3 to 3A when the pituitary adenoma is into the superior cavernous sinus above the ICA and 3B if the pituitary adenoma is into the inferior cavernous sinus below the ICA [10,11]. ICA, intracavernous artery

This classification was revised to divide grade 3 into two subtypes, 3A and 3B, depending on the extension into the inferior or the superior cavernous sinus compartment (Figure 1) [10,11].

All patients with neuro-ophthalmological features of pituitary adenoma underwent transsphenoidal surgery. The extent of tumor resection was classified based on postoperative MRI defining a total and subtotal resection. The complete surgical remission was determined by a 70% reduction of the tumor size at the 6-month MRI.

Histological analysis evaluated the immunostaining of pituitary hormones, including ACTH, prolactin, LH, GH, TSH, and FSH, and the proliferation index (Ki-67) of tissue specimens.

### Statistical analysis

The statistical analysis was performed using the Statistical Package for Social Sciences (SPSS), V21.0. In the descriptive analysis, quantitative variables were normally distributed and presented as means ± SD, and categorical variables were expressed as percentages. The chi-squared test was used to compare categorical variables between independent samples. Statistical significance was considered at *P* < 0.05.

## RESULTS

### Epidemiological and clinical data

**Figure 2:**
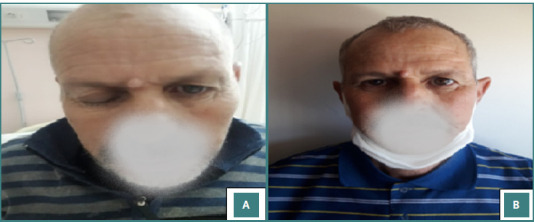
Photos of our patient with NFPA admitted in apoplexy having spontaneous ptosis (A) that improved after surgery (B).

In this report, the prevalence of NFPAs was 30.4%. The mean age was 50.8 ± 11.6 years, and 63.3% of the patients were women with a sex ratio F/M of 1.7. The diagnosis time ranged from 2 to 84 months (median of 5 months). The most common symptom attributed to mass effect was headaches, reported in 93.5% of cases, followed by decreased visual acuity in 80.6%. Seven patients (22.5%) were admitted due to apoplexy, presenting with neuro-ophthalmological symptoms, such as headaches and declining visual acuity. Among these patients, four exhibited ptosis, and one had convergent strabismus (Figure 2). No signs of hormonal hypersecretion were observed on clinical examination. Nevertheless, hypogonadism was the main clinical hyposecretion symptom identified in 16% of patients.

### Biological data

All patients underwent a biological assessment; 13.3 % of patients had partial hypopituitarism, and 66.7% had panhypopituitarism. Central hypogonadism was the main hormonal defect observed in 73.3% of all the cases associated with hyperprolactinemia in 23% of patients. Other hormonal defects were central hypothyroidism (63%), GH deficiency (53%), and central adrenal insufficiency (40%) — one patient presented with diabetes insipidus at the admission for NFPA.

### Radiological data

MRI showed apoplexy in seven patients; there were two patients with microadenoma and 28 patients with pituitary macroadenoma, and 82.5% of those patients had an extracellular extension. Using the Knosp classification, the distribution was as follows: grade 0 in 10% (*n* = 3), grade 2 in 3.3% (*n* = 1), grade 3 in 26.7% (*n* =8), and grade 4 in 60% (*n* = 18). None of the patients had grade 1 of the Knosp classification. The revised classification divided grade 3 into 3A (*n* = 4) and 3B (*n* = 4).

### Knosp classification: correlation with clinical, hormonal profile and surgical remission

Patients with Knosp grade greater than 2 were more symptomatic, with 100% (*n* = 26) (*P* = 0.014) reporting headaches compared to 19 patients reporting a decline in visual acuity (*P* = 0.095). Additionally, GH deficiency was the most common hormonal defect, identified in 61.5% of these patients (*n* = 16) (*P* = 0.037) (Table 1).

**Table 1:** Differences in clinical and hormonal features and surgical remission between invasive and non-invasive NFPAs using Knosp classification

Knosp classification			
	Knosp ≤ 2 (*n* = 4)	Knosp > 2 (*n* = 26)	*P*
Age (years)	60.2 ± 5.3	49.3 ± 11.6	0.080
Female sex	75 (3/4)	61.5 (16/26)	0.530
**Headaches**	50 (2/4)	100 (26/26)	0.014
Decline in visual acuity	25 (1/4)	73.1 (19/26)	0.095
GH deficiency	0	61.5 (16/26)	0.037
Central hypothyroidism	25 (1/4)	69.2 (18/26)	0.126
Adrenal insufficiency	0	46.2 (12/26)	0.112
Central hypogonadism	75 (1/4)	88.5 (23/26)	0.454
Surgical remission	100 (2/2)	42.3 (11/26)	0.206

No significant correlations were found between Knosp classification (grade > 2) and other hormonal deficiencies, such as central hypothyroidism (*P* = 0.126), adrenal insufficiency (*P* = 0.112), and central hypogonadism (*P* = 0.454). All patients with a Knosp grade below 2 achieved complete surgical remission compared to 42.3% for grades beyond 2 (*P* = 0.206) (Table 2).

**Table 2:** Differences in clinical and hormonal features and surgical remission between invasive and non-invasive NFPAs using revised Knosp classification

Revised Knosp classification			
	Knosp ≤ 3A (*n* = 8)	Knosp ≥ 3B (*n* = 22)	*P*
Age (years)	54.5 ± 9.8	49.4 ± 12.1	0.301
Female sex	62.5 (5/8)	63.6 (14/22)	0.637
**Headaches**	75 (6/8)	100 (22/22)	0.064
Decline in visual acuity	62.5 (5/8)	68.2 (15/22)	0.548
GH deficiency	25 (2/8)	63.6 (14/22)	0.071
Central hypothyroidism	50 (4/8)	68.2 (15/22)	0.310
Adrenal insufficiency	37.5 (3/8)	40.9 (9/22)	0.604
Central hypogonadism	75 (6/8)	90.9 (20/22)	0.284
Surgical remission	100 (6/6)	36.4 (8/22)	0.008

Regarding the revised Knosp classification, we did not observe significant differences in clinical and hormonal profiles, except for a tendency toward a higher risk of headaches and GH deficiency in patients with invasive NFPAs (grade ≥ 3B) compared to non-invasive NFPAs (grade ≤ 3A) (100% vs. 75%, *P* = 0.064; 63.6% vs 25%, *P* = 0.071). However, complete surgical remission was achieved in all patients classified as Knosp ≤ 3A (100% vs 36.4%, *P* = 0.008) (Table 2).

## DISCUSSION

One of the notable findings from our research is that patients with invasive NFPAs tended to be symptomatic and exposed to preoperative endocrine deficits. Headaches were the main clinical symptom reported by patients with invasive adenoma (Knosp > 2) (*P* = 0.014), and preoperative endocrine assessment revealed that these patients frequently had GH deficiency (*P* = 0.037).

Preoperative clinical and hormonal evaluation of NFPAs seems imperative, and MRI is the gold standard for diagnosing and evaluating sellar tumors. Pituitary adenomas appear as hypo-or isointense on T1-weighted images. In terms of tumor size, macroadenomas have a smaller size (less than 10 mm), while macroadenomas present as large tumors (more than 10 mm) with extra sellar extension. However, the most concerning feature of PAs on MRI is the cavernous sinus invasion commonly associated with a lower rate of surgical remission. The most recent radiological classification is the Knosp classification, described for the first time by Knosp and others in 1993, defining four grades of local invasion from grade 1 to grade 4, revised in 2015 to differentiate between the inferior and superior invasion of the cavernous sinus in grades 3A and 3B [10,11]. Considering these radiological classifications, our findings were similar to another study conducted by Sanchez *et al*., which showed that larger adenomas (2.4 cm vs. 2.1 cm) had significantly more preoperative endocrine deficits (*P* = 0.02), although their study did not specifically consider Knosp classification [17].

Surgery has become the most effective treatment for adenomas, especially for NFPAs. The gold standard in this field is transsphenoidal surgery, especially for treating compressive macroadenomas. However, surgical remission is not always guaranteed. Transsphenoidal surgery of invasive NFPAs is more complex than non-invasive adenomas, mainly due to the para-sellar extension and the consistency of the tumors. Invasive tumors with fibrous consistency are associated with a greater risk of postoperative complications and incomplete surgical remission [18,19]. In contrast, some previous series found that surgical failure is more closely related to cavernous sinus invasion, independent of tumor size [8]. Otherwise, no clear clinical or histological factors can predict whether non-functioning pituitary adenomas will remit after surgery [5,7]. Therefore, a preoperative MRI seems mandatory for diagnosing and evaluating tumor size and invasion degree, which can ultimately improve postoperative remission prognosis.

The most outstanding results in this research were that the preoperative MRI description considering Knosp and revised Knosp classifications could predict surgical outcomes in NFPAs. Indeed, our patients with invasive NFPAs beyond grade 3B achieved a lower rate of gross total remission (36.4% vs. 100%, *P* = 0.008). This fact was similarly observed by Micko *et al*., showing that grade 3A had a higher rate of surgical cure (64%) than grade 3B (33%, *P* = 0.021) and grade 4 (*P* < 0.001) [20]. It was also in accordance with the results reported by Araujo-Castro *et al*., showing that the rate of surgical remission was lower in invasive PAs than in non-invasive PAs (28,8% vs. 83.1%, *P* < 0.0001) and that the rate of remission decreased as the Knosp grade increased (*P* < 0.001) [8]. Considering these data, the rates of complete surgical cure according to the revised Knosp classification are higher in grades 2 and 3A than those with high-grade adenomas (grades 3B and 4). Those results confirm that the revised Knosp classification appeared more detailed, and the subdivision of grade 3 to 3A and 3B had superiority in predicting surgical remission compared to the Knosp classification grades.

This study has several limitations. First, this was a single-center research with a retrospective analysis of a rare entity of pituitary adenomas, NFPAs, explaining our small patient size and the limitations of our results. Second, radiological definitions of invasiveness may overestimate invasive NFPAs. Therefore, extending our data from patients who are followed at other centers may be difficult.

## CONCLUSION

To accurately predict postoperative surgical cure and assess patients' clinical and hormonal status with non-functioning pituitary adenomas, describing their cavernous sinus invasion using Knosp and revised Knosp classifications is crucial. While NFPAs are hormonally inactive and not linked to hormonal hypersecretion, these classifications remain essential in determining clinical status and surgical remission. Likewise, our report confirms the revised Knosp classification's superiority compared to the Knosp classification in predicting surgical remission.

## Data Availability

Further data is available from the corresponding author upon reasonable request.

## Personal thanks

The authors would like to acknowledge all medical and paramedical staff involved in the management of the patients and the patient’s families for their cooperation and patience.
